# Distribution and seasonal abundance of *Biomphalaria* snails and their infection status with *Schistosoma mansoni* in and around Lake Tana, northwest Ethiopia

**DOI:** 10.1038/s41598-022-21306-0

**Published:** 2022-10-12

**Authors:** Tamirat Hailegebriel, Endalkachew Nibret, Abaineh Munshea

**Affiliations:** 1 Department of Biology, College of Science, Bahir Dar University, Bahir Dar, Ethiopia; 2 Institute of Biotechnology (IOB), Bahir Dar University, Bahir Dar, Ethiopia

**Keywords:** Zoology, Diseases

## Abstract

*Biomphalaria* snails, namely *B. pfeifferi* and *B. sudanica,* are the principal intermediate hosts for *Schistosoma mansoni* infection in Ethiopia. Epidemiological studies of *Biomphalaria* snails and their infection status with *S. mansoni* is vital for public health planning. This study aimed to assess the spatial and seasonal abundance of *Biomphalaria* snails as well as their infection status with *S. mansoni* around Lake Tana, northwest Ethiopia. Malacological survey was conducted from January 2021 to December 2021 in ten different collection sites in and around Lake Tana. Snail collection was performed for 20 min from each collection site seasonally (four times in a year) using a standard scoop and handpicking from aquatic vegetation. All collected snails were carefully examined based on their morphological features and all live *Biomphalaria* snails were subjected to cercariae shedding experiment. Descriptive statistics were used to determine the prevalence of *S. mansoni* infection and its relationship with snail collection sites and seasons. A total of 3886 freshwater snails were collected from ten collection sites around Lake Tana. Out of the total snails collected, 1606 (41.3%; 95% CI 39.77–42.89%) were *Biomphalaria* spp. The highest (374) and the lowest numbers (98) of *Biomphalaria* snails were collected from Shinne River and Qunzela Lakeshore, respectively. Out of the 1375 live *Biomphalaria* snails, 14.4% (95% CI 12.59–16.37%) snails shed cercariae, but only 4.87% (95% CI 3.79–6.15%) were cercariae of *S. mansoni*. The infection prevalence of *S. mansoni* ranged from 10.59% at the Cherechera site to 1.49% at Gumara River. *Biomphalaria* snail infections with *S. mansoni* cercariae were observed throughout the season, the highest and the lowest infection rates being in the spring and summer seasons. Significant differences in the prevalence of *S. mansoni* infection in *Biomphalaria* snails were observed across study sites and seasons (*p* < 0.05). *Biomphalaria* snails were the most abundant freshwater snails found in nearly all of snail collection sites throughout the year. It was revealed that nearly five percent of *Biomphalaria* snails were infected with *S. mansoni* cercariae. This study highlights the importance of appropriate snail control strategies to support the ongoing prevention and control of schistosomiasis around Lake Tana.

## Introduction

Schistosomiasis is one of the neglected tropical diseases (NTD) that is widely distributed in Africa, South America, the Middle East and Southeast Asia^1,2^. The prevalence of the disease varies among regions depending on the socio-economic level, environmental conditions, human water contact behaviour of the community as well as on the level of control strategies employed in the country. The disease is severe in Africa, particularly in sub-Saharan Africa, due to the suitability of the climatic condition and socio-economic development of the region. It is estimated that 85–95% of the global schistosomiasis are in sub-Saharan Africa with the highest prevalence among school-aged childre^3,4^ In Ethiopia, the prevalence of schistosomiasis could reach as high as 90% in some localities, particularly for *Schistosoma mansoni*^5^. Although Ethiopia launched a school-based deworming program in 2015 to control schistosomiasis^6^, the prevalence of the disease is still high in several localities^7–10^.

*Schistosoma mansoni* uses freshwater snails of the genus *Biomphalaria* as an intermediate host to complete its life cycle^11^. Malacological studies have indicated the presence of several snail groups in Ethiopia. *Biomphalaria* species, namely *B. pfeifferi* and *B. sudanica,* serve as intermediate hosts for *S. mansoni*^12,13^ while *Bulinus* snails serve as intermediate hosts for *S. haematobium*^14,15^. *B. pfeifferi* and *B. sudanica* are the principal intermediate hosts for *S. mansoni* in Ethiopia. However, limited information is available about the distribution, abundance and diversity of these snails in several endemic foci of the country. The distribution of schistosomiasis in any endemic foci is directly correlated with the distribution of snail vectors^16,17^. Information regarding the distribution and abundance of *Biomphalaria* snails around Lake Tana is dated back to the beginning of the 1990s^18^. An updated and in-depth investigation of snail intermediate hosts of *S. mansoni* is vital to designing cost-effective snail control strategies in the area.

The infection prevalence of *Biomphalaria* snails with *S. mansoni* varied from 3^19^ to 58%^20^ in Ethiopia. Our previous review showed that about 15% of *Biomphalaria* snails of Ethiopia were positive for *S. mansoni* cercariae^21^. However, the infection status of *Biomphalaria* snails around Lake Tana has not been investigated. A recent evidence showed a high prevalence (35%) of *S. mansoni* infection in humans^7^ in the study area despite the ongoing deworming program. In the present study it was hypothesized that there might be a high-level of *Biomphalaria* snails infected with *S. mansoni* around Lake Tana. Knowing the infection status of freshwater snails with *S. mansoni* serves as one of the important criteria to determine the transmission dynamics of *S. mansoni* in the study area. In addition, assessment of natural snail infection with *S. mansoni* is important to elucidate the level of environmental contaminations with fecal matter from humans as well as from other non-human primates.

Epidemiological studies on the abundance, distribution, and infection status of *Biomphalaria* snails are vital for policymakers to design appropriate schistosomiasis prevention and control strategies. However, there is no recent information on the abundance and distribution of *Biomphalaria* snails and their infection status around Lake Tana. . Therefore, this study aimed to investigate the spatial and seasonal abundance of *Biomphalaria* snails and their infection status with *S. mansoni* in and around Lake Tana, northwestern Ethiopia.

## Material and methods

### Description of the study areas

Lake Tana is located in the north-western part of Ethiopia at 12°0.00' N and 37°0.14' E. Lake Tana is the largest lake in Ethiopia and the major source of the Blue Nile River. The lake consists of more than 37 islands and peninsula and some of them serve for human habitation^22^. Lake Tana covers an area of 3020 km^2^ and a maximum depth of 15 m. Lake Tana is rich in biodiversity with several species of birds, fish, amphibians, macro-invertebrates, and micro-invertebrates. The lake and islands on the lake serve as homes for several species of birds including the endemic ones. Lake Tana consists of 28 known species of fish, of which 68% of them are endemic^23^. As a result of its rich biodiversity, the United Nations Educational, Scientific and Cultural Organization (UNESCO) recognized Lake Tana as a Biosphere reserve in 2015^24^. The present study was conducted at different sites of Lake Tana shores (Dek, Cherechera, Gorgora, Zegie and Qunzela) and tributary rivers of Lake Tana (Enferanz, Gumara, Garno, Shinnie and Robit). The selection of the collection sites was based on human habitation and the frequent human-water contact behaviour of the community.

### Operational definitions of words or phrases

Winter is a dry season in Ethiopia that span from December to February. Spring is span from March to May. There may be occasional rain in most parts of Ethiopia. Summer is the major rainy season in Ethiopia that span from June to August. Autumn is the major harvesting season in Ethiopia that spans from September to November.

### Study design

**Figure 1:**
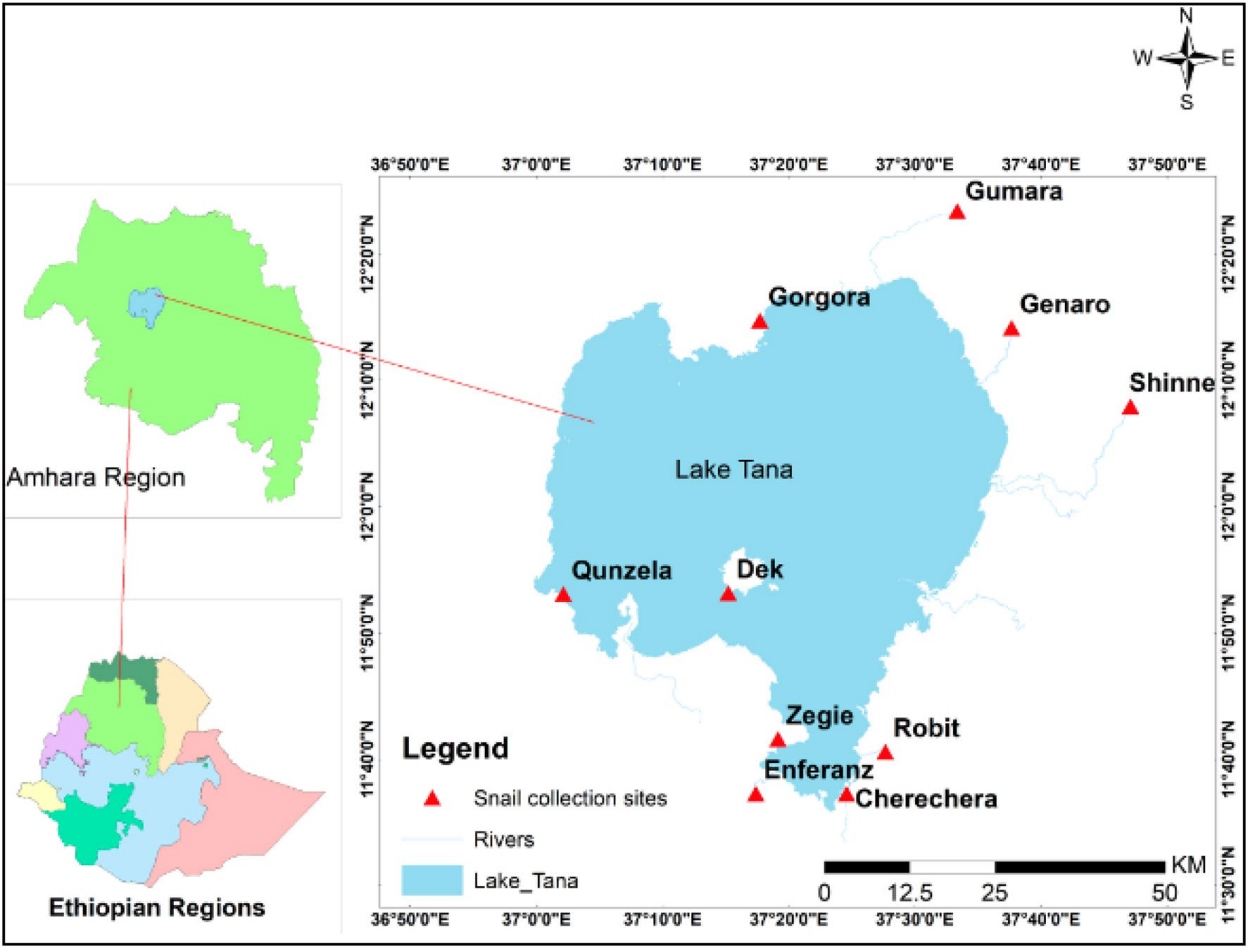
Map of the study areas around Lake Tana. The map was prepared using ArcGIS online software.

Malacological surveys were conducted from January 2021 to December 2021 to assess the distribution and seasonal abundance of *Biomphalaria* snails from the shorelines of Lake Tana (Dek, Cherechera, Gorgora, Zegie and Qunzela) and its tributary rivers, namely Enferanz, Gumara, Garno, Shinnie and Robit (Fig. 1). From each site, snails were sampled from two different points at least 200 m apart. The specific sample collection sites were selected based on the frequency of human-water contact during water fetching, washing clothes, bathing, swimming, fish processing and other domestic activities. The geographical coordinate of each sampling site was taken using a global positioning system (GPS) and it was properly recorded.

### Snail collection

Freshwater snails were collected and examined using a standard protocol as described elsewhere^25^. Snails were sampled using standard scoops (2 mm mesh size) and forceps from water bodies or picked with gloved hands from aquatic vegetation at the shoreline of Lake Tana as well as from the rivers that fed the lake. The snail collection sites were selected based on close proximity to human settlement and high level of open defecation. The scooping was performed for 20 min from each site between 8:00 AM and 10:00 AM on a seasonal basis (four times a year) by the same individual. Samplings were conducted in areas about 10 m along the shorelines of Lake Tana, selected rivers, and from an area of ca.5m^2^ from lake water at each sampling point. Each collected snail was kept separately in a wide-mouth glass bottle filled with water and aquatic vegetation from the same area. The snail samples were transported to the Biomedical Sciences Laboratory of the Department of Biology, Bahir Dar University. All collected snails were sorted, counted and identified in the laboratory.

### Morphological identification of Biomphalaria snails

All the collected snails were carefully examined based on morphological features using standard identification keys to at least a genus level as described elsewhere^25–27^. The common criteria to distinguish snail species include shell shape, shell size, nature of aperture, color and banding pattern of the shell^28^. Once the morphological identification was completed, the snails were kept at dark for 48 h and then they were used for cercariae shedding experiment.

### Testing of snails for S. mansoni infections and identification

Individual snails were carefully transferred into shedding vials that contained 10 ml of natural spring water^29^ with a neutral pH. The shedding of *S. mansoni* and other trematodes cercariae was induced by exposure to artificial light (60 watts) for about two hours at room temperature in the morning (10:00–12:00 AM^30^. Each snail was observed under a dissecting microscope to determine the presence of shedding trematodes cercariae. The water in the shedding vial was carefully examined for the presence of cercariae using a dissecting microscope.

Live cercariae shedding from each snail were transferred to a microscopic slide and covered with a coverslip. The cercariae were carefully observed using a microscope with 40 × magnification power and identified based on their morphological features using a standard identification key^31–33^. The types and the numbers of cercariae discharged from each snail were properly recorded.

### Data analysis

The data generated during the study were analysed using SPSS version 23. Descriptive statistics was used to determine the proportion of *Biomphalaria* snails and their infection prevalence across study sites and study seasons. Analysis of variance (ANOVA) was used to determine the differences in the abundance of *Biomphalaria* snails across study sites and seasons. Chi-square test was used to asssess the relationship between *Biomphalaria* snail infection with *S. mansoni* and studies sites and seasons. The geographic coordinate of snail collection sites was taken using a global positioning system (GPS) from each sampling point. Mapping of the snail collection site was prepared using ArcGIS online free software. For all statistical analyses, a p-value below 0.05 was used to declare statistical significance..

### Ethics approval and consent to participate

This study was conducted after obtaining ethical clearance from the Ethical Review Committee of College of Science, Bahir Dar University with Ref. No. PGRCSVD/155/2020. The objective of the study was explained to the local administration before snail collection.

## Results

**Figure 2:**
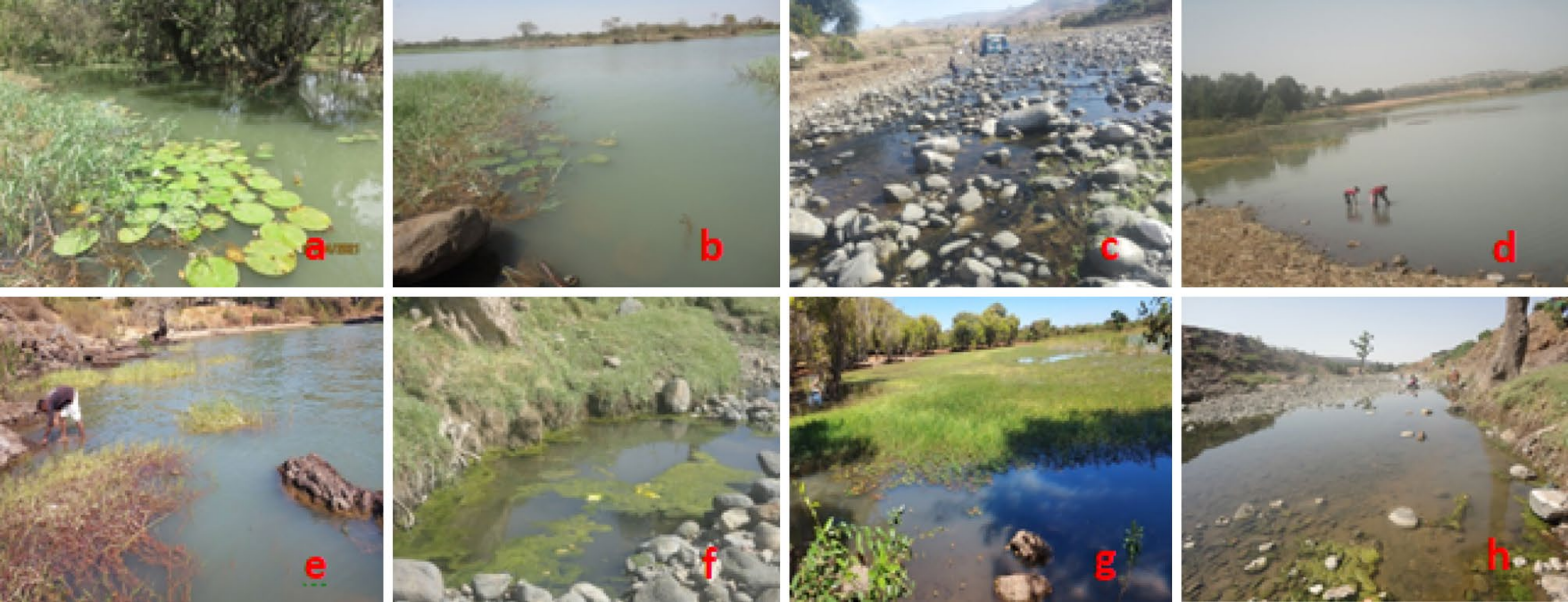
Image taken from some of the snail collection sites around Lake Tana; (**a**) Enferanz river (**b**) Cherechera site (**c**) Garno river (**d**) Qunzela port (**e**) Gorgora port (**f**) Shinne river (**g**) Dek Island (**h**) Robit river. All the images were taken by the corresponding author (TH).

The study was conducted at a latitudinal range of 11.6181–12.3909° E and a longitudinal range of 37.0343–37.7848°N. The collection sites were classified as periphery of Lake Tana and its tributary rivers. All snails were collected from the area where there is frequent human-water contact for various activities such as washing clothes, bathing, swimming, fetching water and fish processing (Table 1). The snail collection sites had either muddy or stony substrates with clean or turbid water. Some of the images of snail collection sites are presented in Fig. 2.

**Table 1:** Sampling points, GPS coordinates and other basic information of the snail collection sites.

Study area	Sampling point	Elevation (masl)	GPS coordinate		Human activity	Nature of substrate	Nature of the water	Vegetation type	Habitat classification
			Latitude	Longitude					
Cherechera	a	1793	11.62069	37.40997	Swimming, bathing and fish processing	Muddy	Turbid water	Floating vegetation	Lake periphery
	b	1789	11.61805	37.41066					
Dek Island	a	1787	11.88602	37.25337	Bathing, washing cloth & fish processing	Muddy	Turbid water	Papyrus and other floating vegetation	Lake periphery
	b	1788	11.88646	37.25124					
Zegie Peninsula	a	1787	11.69279	37.31926	Swimming, bathing and washing cloth	Rocky	Clear	Floating vegetation	Lake periphery
	b	1782	11.69217	37.31730					
Qunzela town	a	1806	11.88451	37.03577	Bathing, fetching & washing cloth	Muddy	Turbid water	Floating vegetation	Lake periphery
	b	1790	11.88241	37.03426					
Gorgora Peninsula	a	1802	12.24575	37.29543	Bathing, washing cloth & fish processing	Rocky	Clean	Floating vegetation	Lake periphery
	b	1790	12.24256	37.29681					
Enferanz river	a	1820	11.62090	37.28981	Fetching and washing cloth	Muddy	Turbid water	Floating vegetation	River
	b	1807	11.62208	37.28934					
Shinie river	a	1957	12.13247	37.78487	Bathing & washing cloth	Rocky	Clean water	Covered with green algae	River
	b	1950	12.13083	37.78442					
Robit river	a	1849	11.67600	37.46081	Bathing & washing cloth	Rocky	Clean water	Algae & floating vegetation	River
	b	1841	11.67606	37.45956					
Gumara river	a	1906	12.39091	37.55574	Bathing, fetching & washing cloth	Rocky	Turbid water	Covered with algae	River
	b	1903	12.39083	37.55716					
Garno river	a	1859	12.23636	37.62763	Bathing and washing cloth	Rocky	Clean water	Covered with algae	River
	b	1856	12.23685	37.62878

### The abundance of freshwater snails in Lake Tana and tributary rivers

**Figure 3:**
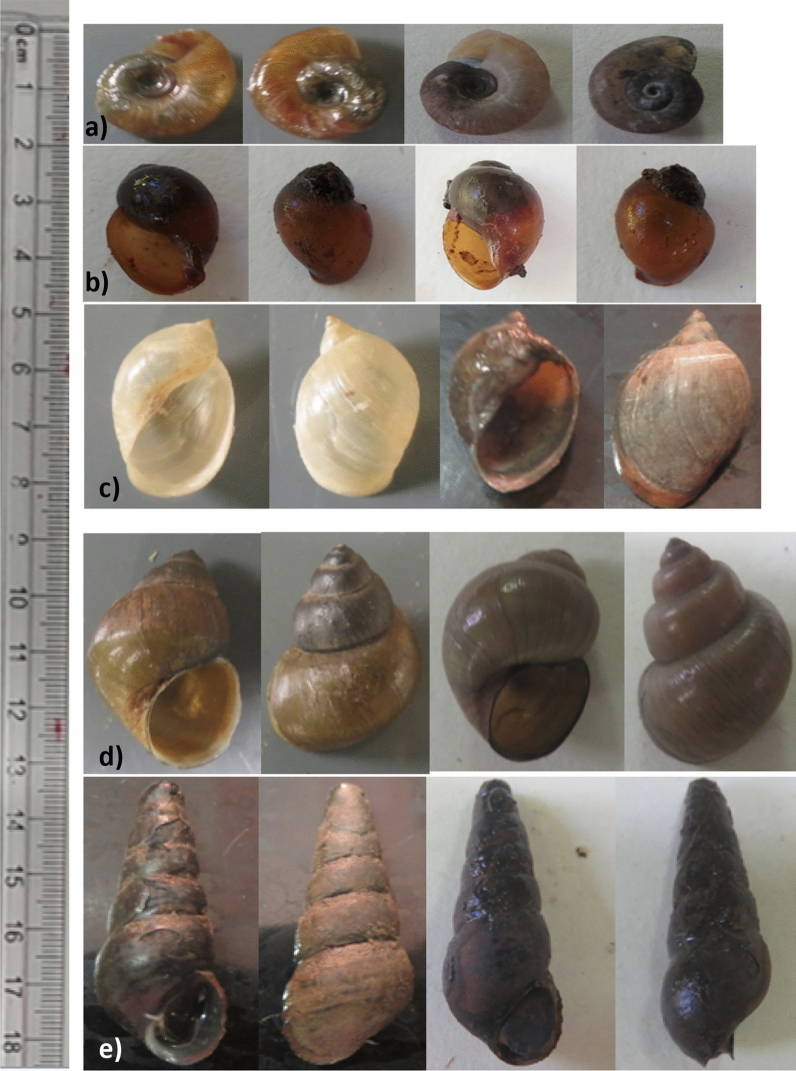
Freshwater snail genera collected from Lake Tana; (**a**) ***Biomphalaria*** snails (right and left side view), (**b**) ***Bulinus*** snails (apertural and abapertural view), (**c**) ***Lymnaea*** snails (ventral and dorsal view), (**d**) ***Bellamya*** snails (ventral and dorsal view) and (**e**) ***Melanoides*** snails (ventral and dorsal view). All the images were taken by the corresponding author (TH).

A total of 3886 freshwater snails were collected from 20 sampling points at ten study sites during the study period. Five freshwater snail genera, namely *Biomphalaria, Lymnaea, Bulinus*, *Melanoides* and *Bellamya* snails, were recorded from the study areas (Fig. 3). The dominant snail genus observed in the study area was *Biomphalaria* (41.33%, 95% CI 39.77–42.89%) followed by *Lymnaea* (Table 2). *Lymnaea* snails were identified from all snail collection sites while *Biomphalaria* snails were observed from nine snail collection sites. *Melanoides* and *Bellamya* were observed from limited study sites, mainly from the Lake Tana periphery.

**Table 2:** Diversity and abundance of freshwater snails of Lake Tana and its tributary rivers, 2021/22.

Collection sites	Freshwater snail Genus observed in the study area					
	*Biomphalaria* snails No. (%)	*Bulinus* snails No. (%)	*Lymnaea* snails No. (%)	*Melanoides* snails No. (%)	*Bellamya* snails No. (%)	Total No. (%)
Cherechera	194 (12.1)	87 (19.6)	40 (3.0)	139 (70.2)	71 (23.0)	531 (13.70)
Dek Island	173 (10.8)	119 (26.8)	151 (11.4)	0	34 (11.0)	477 (12.3)
Zegie Peninsula	99 (6.2)	65 (14.6)	63 (4.7)	37 (18.7)	76 (24.6)	340 (8.7)
Qunzela town	98 (6.1)	68 (15.3)	173 (13.0)	9 (4.6)	58 (18.8)	406 (10.5)
Gorgora Peninsula	0	17 (3.8)	56 (4.2)	0	38 (12.3)	111 (2.8)
Enferanze river	128 (7.9)	45 (10.1)	168 (12.6)	12 (6.1)	32 (10.4)	385 (9.9)
Shinie river	374 (23.3)	0	240 (18.1)	0	0	614 (15.8)
Robit river	240 (14.9)	42 (9.5)	319 (24.0)	0	0	601 (15.5)
Gumara river	162 (10.1)	1 (0.23)	60 (4.5)	0	0	223 (5.7)
Garno river	138 (8.6)	0	59 (4.4)	1 (0.5)	0	198 (5.1)
All sites	1606 (41.33)	444 (11.43)	1329 (34.20)	198 (5.10)	309 (7.94)	3886 (100)

### Spatial and seasonal abundance of Biomphalaria snails

**Figure 4:**
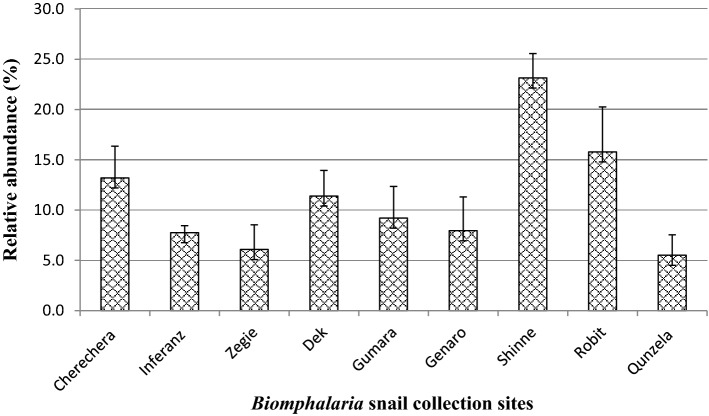
Relative abundance of *Biomphalaria* snails at different collection sites.

**Figure 5:**
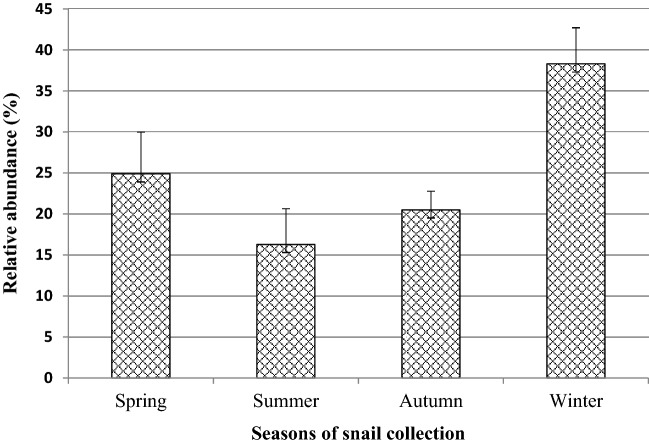
Relative abundance of *Biomphalaria* snails on a seasonal basis.

*Biomphalaria* snails were collected from 18 sampling points around Lake Tana. A total of 1606 *Biomphalaria* snails were collected from the nine study sites around Lake Tana. The highest (23.29%; 95% CI 21.2–25.4%) and the lowest (6.1%; 95% CI 4.9–7.4%) number of *Biomphalaria* snails were collected from Shinne River and Qunzela lakeshore, respectively (Fig. 4). Similarly, the abundance of *Biomphalaria* snails varied across snail collection seasons. The seasonal distribution showed that the winter season had the highest *Biomphalaria* snail abundance, 36.43% (95% CI 34.07–38.83%), while the summer season showed the lowest abundance of *Biomphalaria* snail, 18.06% (95% CI 16.21–20.03%) (Fig. 5). There was a significant difference in the abundance of *Biomphalaria* across study sites and seasons (*p* < 0.05).

### Comparison of Biomphalaria snails by habitat

**Figure 6:**
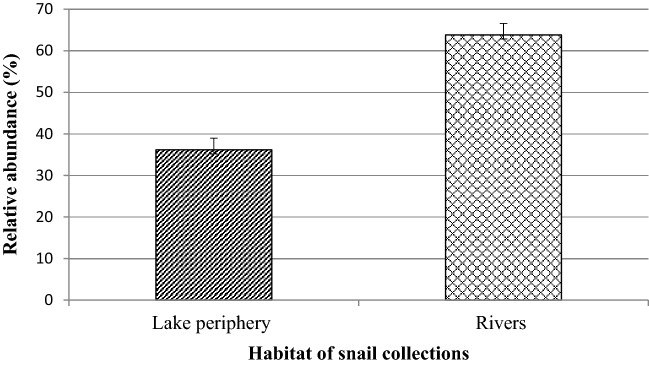
Relative abundance of *Biomphalaria* snails at study habitat.

Freshwater snails were collected from two types of habitats: tributary rivers and Lake periphery. *Biomphalaria* snails were more common in rivers with a sandy and stony basement than in lakeshores (Fig. 6). There was a significant difference in the number of *Biomphalaria* snails between the lakeshore and riverine areas (*p* = 0.026).

### Infection status of Biomphalaria snails of Lake Tana and its tributary rivers

**Figure 7:**
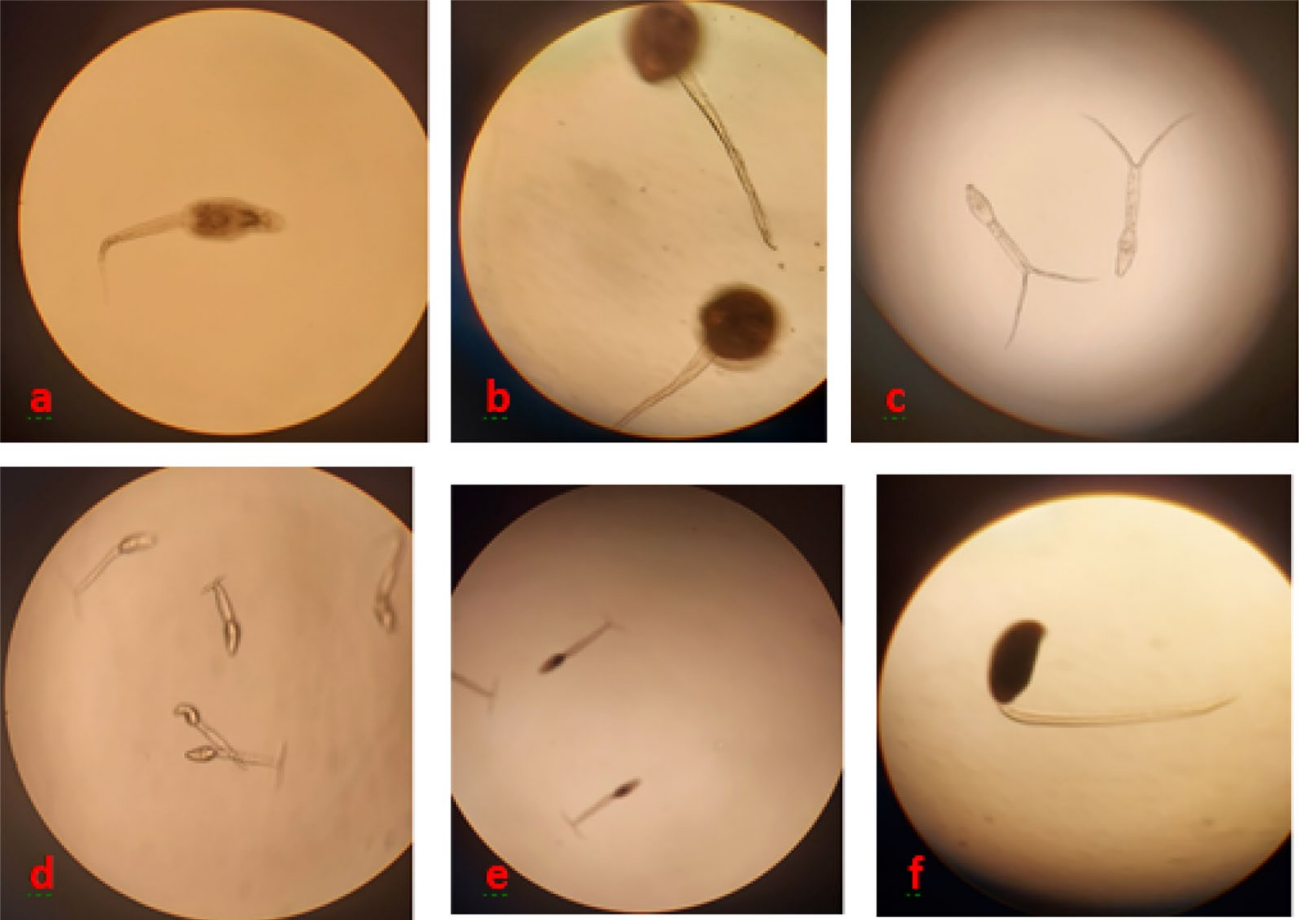
Trematode cercaria shed by *Biomphalaria* snails 100 × magnification; (**a**) *Echinostome cercaria*, (**b**) *Amphistome cercaria*, (**c**) *Brevifurcate-apharyngeate diastome cercaria*, (**d, e**) *Schistosome cercaria,* (**f**) Unidentified cercaria. *Echinostome* and *Schistosome* cercaria observed from all study sites while *Brevifurcate diastome* cercariae were observed in Cherechera, Enferanze, Robit and Dek. Unidentified cercaria was observed from Dek Island.

A total of 1375 live *Biomphalaria* snails were tested for trematode infection. Among these snails, 14.40% (95% CI 12.59–16.37%) snails shed trematode cercariae, but only 4.87% (95% CI 3.79–6.15%) were cercariae of *S. mansoni* (Table 3). The common trematodes observed in this study consisted of cercariae of *Schistosoma mansoni, Amphistome, Echinostome, Brevifurcate apharyngeate distome* and unidentified cercaria (Fig. 7).

**Table 3:** Prevalence of *Schistosoma mansoni* and other trematodes infection among *Biomphalaria* snail species.

Snail collection sites	Snail count	Snail examined for cercaria	*S. mansoni* cercaria	Other trematodes cercaria	Total trematodes cercaria
	Number	Number	Number (%)	Number (%)	Number (%)
Cherechera	194	170	18 (10.59)	23 (13.53)	41 (24.12)
Inferanz river	128	109	6 (5.50)	24 (22.02)	30 (27.52)
Zegie Peninsula	99	84	2 (2.38)	6 (7.14)	8 (9.52)
Dek Island	173	143	7 (4.90)	19 (13.29)	28 (19.58)
Gumara river	162	134	2 (1.49)	6 (4.48)	8 (5.97)
Genaro river	138	127	2 (1.57)	12 (9.45)	14 (11.02)
Shinne river	374	313	18 (5.75)	23 (7.35)	41 (13.10)
Robit river	240	206	6 (2.91)	12 (5.83)	18 (8.74)
Qunzela town	98	89	6 (6.74)	4 (4.49)	10 (11.24)
Total	1606	1375	67 (4.87)	129 (9.38)	198 (14.40)

**Figure 8:**
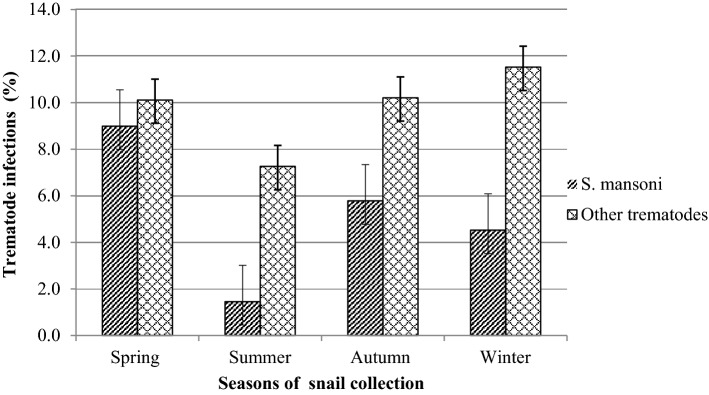
Seasonal variations of *S. mansoni* and other trematodes infection.

The highest *S. mansoni* infection was observed from the Cherechera site (10.59%) followed by Qunzela site (6.74%) while the lowest *S. mansoni* infection was observed from the Gumara River (1.49%). A significant difference in the infection prevalence was observed across study sites (*p* = 0.004). The study was conducted in all seasons and the highest and the lowest *S. mansoni* cercariae were observed during the spring and summer seasons, respectively (Fig. 8). There was a significant difference in the infection prevalence of *Biomphalaria* snails across study seasons (*p* < 0.001).

## Discussion

Epidemiological studies about the snail intermediate snail host species are vital for policymakers to design appropriate schistosomiasis control strategies. The principal intermediate host for *S. mansoni* in Ethiopia is *Biomphalaria* species^12,13^. Assessment of abundance, distribution and infectiction status of *Biomphalaria* snails contributes a lot to the prevention and control of schistosomiasis in the country. Schistosomiasis control strategies might not be effective without considering the snail intermediate hosts. In line with this, the present study aimed to determine the abundance, distribution and infection status of *Biomphalaria* snails with *S. mansoni* cercariae in and around Lake Tana.

The present study was conducted at lakeshores and tributary rivers of Lake Tana.This study revealed the presence of *Biomphalaria, Bulinus, Lymnaea, Melanoides* and *Bellamya* snails in the study sites. Among these snail genera, *Biomphalaria* snail was the predominant snail genus and it was recorded from nine study sites, which is in agreement with reports from studies conducted at Gibe River Basin, Ethiopia^34^, Kenya and Tanzania^35^. *Biomphalaria* snails were more common in rivers than in the lakeshores, which is in line with reports from studies conducted in Ethiopia^36^, East Africa^37^, South Africa^38^, Nigeria^39^ and Kenya^40^. This shows that *Biomphalaria* snails prefer rivers and streams that have clear water with sandy and gravel substrates to lakeshores that have muddy substrates.

### Seasonal variation of Biomphalaria snails

The abundance and distribution of *Biomphalaria* snails varied significantly across study seasons. *Biomphalaria* snails were dominant during winter and spring as compared with other seasons. Similar observations were reported from Egypt^41^. Several studies have shown that the abundance of *Biomphalaria* snails was higher in the dry season than in wet season^42–44^. In contrast to our finding, *Biomphalaria* snails were more abundant during the wet seasons than in the dry season in South Africa^29^. This might be associated with the water temperature, velocity, turbidity and other environmental parameters of the study area. High rainfall, water velocity, and turbidity during the rainy season affect the natural habitats of snails in Ethiopia. As a result of these environmental conditions, the abundance of *Biomphalaria* snails may decline in the study area. This suggests that *Biomphalaria* snails may prefer stable habitat for survival.

### The spatial variation of Biomphalaria snails

The abundance of *Biomphalaria* snails varied across study sites. *Biomphalaria* snails were recorded from all study sites except Gorgora. Although we attempted several times to search for *Biomphalaria* snails from Gorgora peninsula, we could not find *Biomphalaria* snails. The abundance of *Biomphalaria* snails varied from 6.1% to 23.3% in the different study sites. Spatial variations in the abundance of *Biomphalaria* snails across study sites were well documented^35,40,45,46^. It is known that snail abundance varies from area to area depending on different environmental and biotic factors. In the present study, the difference in abundance of snails across sites might be associated with the nature of study sites, *Biomphalaria* snails being more abundant in rivers than in lakeshores. A study in Senegal showed that *Biomphalaria* snails preferred clean rivers and streams having stony and gravel substrates^47^. The overall variation in the abundance of *Biomphalaria* snails might be associated with the nature of the water, types of aquatic vegetation, nature of water substrate, geographical locations and other environmental parameters.

### Infection status of Biomphalaria snails

The current study showed that 14.4% of *Biomphalaria* snails shed different types of trematodes cercariae, which is in agreement with reports from studies conducted in Egypt^48^ and Tanzania^49^. In contrast to the present finding, only 4.6% of *Biomphalaria* snails were infected with trematodes around Omo Gibe River Basin in Ethiopia^34^. The present study revealed that 4.87% *Biomphalaria* snails were infected with *S. mansoni*, which is in agreement with reports from studies conducted in different parts of Ethiopia^50,51^ as well as with finding in systematic review and meta-analysis from African countries ^52^. In contrast, high prevalence of *Schistosoma mansoni* cercariae in *Biomphalaria* snails were reported from different parts of Ethiopia^13,20,53^, Tanzania^49,54^ and Nigeria^55^. The proportions of *Biomphalaria* snails infected with schistosome cercariae reported from Kenya were even lower than our findings^40,45^. The difference in infection status of *Biomphalaria* snails observed across studies is mainly linked to the types of diagnostic methods used. Superior detection of *S. mansoni* infection from *Biomphalaria* snails was obtained using PCR compared to cercarial shedding experiments. For example, 12% vs. 47% was reported from Tanzania^49^ and 5% vs. 27% shown in review paper in African countries^52^. In addition, anthropogenic activities, geographical locations, water quality, types of aquatic vegetation and other environmental factors might have contributed to the observed differences.

Significant variation of *Biomphalaria* infection with *S. mansoni* was observed across the study seasons. The highest infection rate was observed during the dry season as compared to the wet season, which is in line with reports from Tanzania^56^, Sudan^43,57^ and Nigeria^58^. High levels of open-field defecation, human-water contact activities, and stable water conditions are observed during the dry seasons of the year in Ethiopia. These conditions might contribute to the long-term survival of *Biomphalaria* snails leading to high chance of infection with *S. mansoni* miracidia.

The infection status of *Biomphalaria* snails varied across study sites. In this study, it was revealed that the proportion of infected *Biomphalaria* snails was higher along the Lake periphery than in rivers. In contrast to this finding, more number of infected *Biomphalaria* snails were reported from lakeshores as compared to rivers and streams in western Kenya^40^. These variations are mainly linked to the level of anthropogenic activities such as human-water contact activities and open-field defecation. High *Biomphalaria* snail infection was observed in the area where there is frequent human- water contact activities associated with washing clothes, swimming, bathing, fetching water and fish processing.

Schistosomiasis control strategies in sub-Saharan African countries including Ethiopia focus on mass-drug administration to school-aged children, with little or no emphasis on snail control. In this study, *Biomphalaria* snails were shown to be sources of *S. mansoni* infection and therefore it is an appropriate area for intervention to support the ongoing schistosomiasis prevention and control in the study area. Therefore, policymakers are advised to revisit the current schistosomiasis control and prevention strategies in Ethiopia.

### Limitation of the study

Water quality and its association with snail abundance were not assessed in the present study. Water quality may have an impact on the abundance of *Biomphalaria* snails at different study sites as well as across seasons. The study was conducted at ten different sites and four seasons making it difficult to collect information for water quality analysis. In the present study, we used a cercarial shedding experiment which has lower sensitivity compared to PCR approaches^49^. This might have led to the underestimation of the true prevalence of *S. mansoni* cercariae in *Biomphalaria* snails.

## Conclusion

Lake Tana and its tributary rivers serve as suitable habitats for freshwater snails particularly for *Biomphalaria* snails. *Biomphalaria* species were abundant freshwater snails and they were present in varied numbers in nearly all of snail collection sites throughout the year. In this study it was revealed that nearly five percent of *Biomphalaria* snails were infected with *S. mansoni* cercariae. The prevention and control of schistosomiasis in Ethiopia totally rely on mass-drug administration without giving due consideration to snail control. This study showed that *Biomphalaria* snails were important sources of *S. mansoni* infection to humans living in the nearby snail habitats. Therefore, policymakers, regional administrators, and other stakeholders working on schistosomiasis need to incorporate appropriate snail control strategies to support the ongoing schistosomiasis prevention and control strategies.

## Data Availability

All data generated or analyzed during this study are included in this published article.

## Abbreviations

IoB: Institute of biotechnology
GPS: Global positioning system
SPSS: Statistical package for social science
MASL: Meter above sea level
NTD: Neglected tropical diseases
UNESCO: United Nations Educational, Scientific and Cultural Organization
PCR: Polymerase chain reaction
CI: Confidence interval
MDA: Mass drug administration
